# Estimation of Serum C-reactive Protein, Nitric Oxide, Superoxide Dismutase, Glutathione Reductase, and Glutathione Peroxidase Level in Lung Cancer

**DOI:** 10.7759/cureus.100448

**Published:** 2025-12-30

**Authors:** Sushant S Dhanavade, Karan Pujari

**Affiliations:** 1 Biochemistry, Krishna Institute of Medical Sciences, Karad, IND; 2 Biochemistry, Government Medical College and Hospital, Ratnagiri, Ratnagiri, IND

**Keywords:** chromosome 21, c-reactive protein, glutathione peroxidase (gpx), glutathione reductase, interleukin – 1 β, lung cancer, nitric oxide (no)

## Abstract

Background

Oxidative stress and systemic inflammation play critical roles in lung cancer progression. Biomarkers, such as C-reactive protein (CRP), nitric oxide (NO), and key antioxidant enzymes, may contribute to understanding the disease mechanism.

Objective

The objective of this study is to evaluate serum levels of CRP, NO, superoxide dismutase (SOD), glutathione reductase (GR), and glutathione peroxidase (GPx) in lung cancer patients compared with healthy controls.

Methods

A case-control study was conducted involving 50 lung cancer patients and 50 age- and sex-matched healthy controls. Serum CRP, NO, SOD, GR, and GPx levels were analyzed using standard biochemical methods, including turbidimetric immunoassay and spectrophotometric techniques. Statistical analysis was performed using SPSS version 26 (IBM Corp., Armonk, NY). A p-value < 0.05 was considered statistically significant.

Results

Significantly increased serum CRP and NO levels and significantly reduced SOD, GR, and GPx enzyme activities were observed among lung cancer patients compared with controls (p < 0.001). No significant correlations were identified between inflammatory and antioxidant biomarkers.

Conclusion

The imbalance between elevated inflammatory markers and reduced antioxidant defense suggests enhanced oxidative stress in lung cancer patients. These biomarkers may support clinical assessment and disease monitoring.

## Introduction

Lung cancer persists as the most lethal malignancy worldwide, accounting for more than 1.8 million annual deaths. Its poor prognosis is attributed to late presentation, frequent metastasis, and limited therapeutic responsiveness. Carcinogenesis in lung cancer is driven by genetic mutations, chronic inflammatory responses, environmental carcinogens, and oxidative stress-mediated cellular damage [1].

Inflammation is now recognized not only as a consequence but also as a causative factor in cancer. Pro-inflammatory cytokines such as IL-6 stimulate hepatic synthesis of CRP, which has been repeatedly shown to correlate with tumor progression, lymph node involvement, cachexia, and reduced survival. Therefore, CRP functions as a simple and inexpensive systemic inflammatory biomarker [2].

Nitric oxide (NO), a reactive nitrogen species produced by inducible NO synthase (iNOS), plays dual roles in cancer biology. While physiological levels maintain vascular integrity and immune function, excessive NO leads to mutagenesis, promotes angiogenesis, inhibits apoptosis, and facilitates tumor metastasis. Thus, monitoring NO offers insights into tumor microenvironment changes [3].

Oxidative stress is defined as an imbalance between the excessive generation of reactive oxygen species (ROS) and a weakened antioxidant system. Malignant cells typically exhibit heightened oxidative stress due to increased metabolic rate, mitochondrial dysfunction, and inflammatory cytokine signaling. Antioxidant enzymes such as SOD, GR, and GPx are vital in neutralizing ROS and mitigating oxidative DNA damage. Their depletion compromises cellular defense, allowing mutational accumulation and increased malignancy [4].

Recent literature suggests oxidative markers are not merely bystanders but active drivers of lung cancer progression, treatment resistance, and recurrence. However, the combined evaluation of both oxidative and inflammatory biomarkers in lung cancer patients remains underexplored in many regional populations [5]. Although previous research supports the role of oxidative and inflammatory biomarkers in lung cancer development, findings remain varied, and clinical applicability continues to evolve. Therefore, the present study was conducted to compare serum levels of CRP, NO, SOD, GR, and GPx between lung cancer patients and healthy controls and to assess whether a relationship exists between inflammatory and oxidative stress markers.

## Materials and methods

Study design and ethical approval

This was a retrospective case-control study conducted in the Department of Biochemistry, Government Medical College, Miraj, Maharashtra, India. The study was conducted from January 2018 to December 2020. Ethical approval was obtained from the Ethics Committee, Government Medical College, Miraj, before study initiation, and written informed consent was taken from each participant.

Study population

Fifty clinically and histopathologically confirmed lung cancer patients (Group I) and 50 apparently healthy age- and sex-matched controls (Group II) were included. Diagnosis was based on clinical evaluation, chest computed tomography (CT) imaging, and histopathology according to the standard WHO Classification of Thoracic Tumours (5th edition) [6].

Inclusion and exclusion criteria

Eligible participants were those newly diagnosed and treatment-naïve for lung cancer, aged > 40 years. Exclusion criteria included diabetes mellitus, cardiovascular diseases, renal or liver disorders, inflammatory or infectious diseases, other active malignancies, and recent use of corticosteroids or antioxidant supplements. Subjects who did not consent were excluded.

Research population

The sample size of 50 participants per group was determined based on feasibility and consistency with effect sizes observed in previous biomarker comparison studies [7,8]. This sample size provided approximately 80% power to detect a statistically significant difference (p < 0.05) in primary antioxidant enzyme levels (SOD, GPx) between groups, assuming a large effect size (Cohen's d > 0.8).

Blood collection and processing

Four milliliters of venous blood were collected under aseptic precautions using a disposable needle and syringe (Hindustan Syringes & Medical Devices Ltd., Haryana, India). Samples were transferred into plain vacutainer tubes (HiMedia Laboratories Pvt. Ltd., Mumbai, India) and centrifuged at 3000 rpm for 10 minutes to obtain clear, non-hemolyzed serum for analysis.

Biochemical analysis

CRP was quantified using a turbidimetric immunoassay kit (Agappe Diagnostics Ltd., Kerala, India; Catalog No: Rev. No.: ADL/IFU/CRP/120FR/R02; ISO 9001:2015 EN ISO 13485:2016). This is a latex-enhanced turbidimetric immunoassay. CRP in the samples binds to specific anti-CRP antibodies, which have been adsorbed to latex particles and agglutinates. The agglutination is proportional to the quantity of CRP in the sample. NO concentration was estimated using the Griess reagent method. Antioxidant enzyme levels of SOD (U/mL), GR (mM NADPH/min/g), and GPx (mM GSH/min/mg) were measured using standard spectrophotometric methods as described in established protocols [9,10].

Statistical analysis

Data were analyzed using SPSS version 26 (IBM Corp., Armonk, NY). Mean ± standard deviation (SD) was used to present continuous variables. An independent samples t-test was applied to compare groups. Pearson’s correlation coefficient was used to assess associations among biomarkers. A p-value < 0.05 was considered statistically significant.

## Results

Baseline characteristics

The study groups were well-matched for age and sex distribution, ensuring comparability for biomarker analysis. The mean age of lung cancer patients (Group I) was 62.14 ± 8.6 years, which was not statistically different from the mean age of healthy controls (Group II) at 61.32 ± 7.9 years (p = 0.58). The male-to-female ratio was 32/18 in Group I and 30/20 in Group II, showing no significant difference (p = 0.67). This successful matching minimizes the potential confounding effects of these demographic variables on the observed biomarker levels (Table 1).

**Table 1:** Baseline Characteristics of Study Participants This table presents the demographic profile of the study cohort, confirming successful matching between lung cancer patients (Group I, n = 50) and healthy controls (Group II, n = 50). Data for age are presented as mean ± standard deviation (SD) and were compared using an independent samples t-test. Sex distribution is presented as counts (male/female) and was compared using the chi-square test. The p-values and test statistics indicate no statistically significant differences between the groups for these baseline variables, validating the case-control matching.

Variable	Group I (n = 50)	Group II (n = 50)	Test Statistic	p-value
Age (years), mean ± SD	62.14 ± 8.6	61.32 ± 7.9	t = 0.56	0.58
Sex (M/F)	32/18	30/20	χ² = 0.18	0.67

Biochemical parameters

Lung cancer patients exhibited a pronounced imbalance in serum biomarkers compared to healthy controls. Inflammatory markers were significantly elevated: mean CRP was nearly five times higher in patients (22.67 ± 6.20 mg/dL) than in controls (4.60 ± 3.50 mg/dL), and mean NO was 62% higher (32.30 ± 7.50 μmol/L vs. 19.90 ± 5.20 μmol/L) (p < 0.001 for both). Conversely, all measured antioxidant enzymes were significantly depleted. SOD activity was reduced by 44% (2.86 ± 0.16 U/mL vs. 5.11 ± 0.60 U/mL), GR by 67% (3.25 ± 0.34 mM NADPH/min/g vs. 9.76 ± 0.43 mM NADPH/min/g), and GPx by 53% (16.52 ± 1.25 mM GSH/min/mg vs. 34.96 ± 6.29 mM GSH/min/mg) (p < 0.001 for all) (Table 2). These results confirm a state of heightened inflammation and oxidative stress in the patient cohort.

**Table 2:** Serum Biochemical Parameters in Lung Cancer Patients and Controls This table summarizes the comparison of key inflammatory and antioxidant biomarkers between the study groups. Data are presented as mean ± standard deviation (SD). Group I consists of lung cancer patients (n = 50), and Group II consists of healthy controls (n = 50). Statistical comparison was performed using an independent samples t-test. The p-values (<0.001) and t-statistics indicate extremely statistically significant differences for all parameters. CRP: C-reactive protein; GPx: glutathione peroxidase; GR: glutathione reductase; GSH: reduced glutathione; NADPH: nicotinamide adenine dinucleotide phosphate; NO: nitric oxide; SOD: superoxide dismutase

Parameter (unit)	Group I mean ± SD	Group II mean ± SD	t-value	p-value
CRP (mg/dL)	22.67 ± 6.20	4.60 ± 3.50	18.45	<0.001
NO (μmol/L)	32.30 ± 7.50	19.90 ± 5.20	9.84	<0.001
SOD (U/mL)	2.86 ± 0.16	5.11 ± 0.60	-26.12	<0.001
GR (mM NADPH/min/g)	3.25 ± 0.34	9.76 ± 0.43	-85.51	<0.001
GPx (mM GSH/min/mg)	16.52 ± 1.25	34.96 ± 6.29	-20.71	<0.001

Correlation analysis

Pearson’s correlation analysis revealed no statistically significant linear relationships (p > 0.05) between the levels of inflammatory markers (CRP, NO) and any of the antioxidant enzymes (SOD, GR, GPx) within the lung cancer patient group.

## Discussion

The findings of this study demonstrate a significant systemic imbalance characterized by elevated inflammation and depleted antioxidant defense in lung cancer patients, consistent with the established paradigm of oxidative stress in carcinogenesis [4].

The marked elevation in serum CRP aligns with its recognized role as a non-specific acute-phase reactant upregulated by pro-inflammatory cytokines like IL-6 in the tumor microenvironment [2]. Our observed mean CRP level of 22.67 mg/dL is substantially higher than values reported in some studies of non-small cell lung cancer [11], potentially reflecting the advanced disease state or high inflammatory burden in our cohort. Similarly, the significant increase in NO supports its proposed pro-tumorigenic functions. Elevated NO, often driven by iNOS overexpression in tumor and stromal cells, can promote DNA damage, angiogenesis, and metastasis, contributing to disease progression [3].

Conversely, the significant depletion of key antioxidant enzymes - SOD, GR, and GPx - suggests a compromised capacity to neutralize reactive oxygen and nitrogen species. This depletion may result from excessive consumption during persistent oxidative insult, downregulation of antioxidant gene expression (e.g., via Nrf2 pathway inhibition), or a combination of both [12]. Our finding of reduced GPx activity is particularly notable, as this enzyme is crucial for detoxifying lipid hydroperoxides and regulating ferroptosis, a form of cell death relevant to cancer [13]. However, it is important to contextualize these findings within the broader literature. Some studies have reported increased levels of certain antioxidant enzymes in tumor tissue or serum as an adaptive response to oxidative stress [14]. This discrepancy may be attributed to differences in cancer stage, histological subtype, or the compartment (tissue vs. serum) being analyzed. Our data from treatment-naïve patients suggest that in systemic circulation, a net depletion of enzymatic antioxidants is evident.

The absence of significant correlation between individual inflammatory and antioxidant markers is an interesting finding. It implies that within the complex pathophysiology of lung cancer, the degree of systemic inflammation (as indicated by CRP and NO) may not directly predict the level of antioxidant depletion in a simple linear fashion. The absence of correlation suggests that within lung cancer, systemic inflammation and antioxidant depletion are not directly linked in a simple linear manner. This is likely because these processes are regulated by independent yet interconnected signaling networks. For example, inflammation is primarily driven by pathways like NF-κB, while antioxidant responses are controlled separately by factors like Nrf2. They function independently but are interconnected through crosstalk molecules like ROS, which can both stimulate inflammation and deplete antioxidants. This complex interaction within the tumor microenvironment explains why individual markers like CRP and antioxidants may not correlate directly.

Clinically, this biomarker profile - high CRP/NO and low SOD/GR/GPx - could serve as a composite signature of oxidative stress burden. It may have utility in risk stratification, monitoring disease progression, or evaluating response to therapies that modulate oxidative stress or inflammation [15]. For instance, rising antioxidant levels during treatment could indicate a positive systemic response.

Limitations

This study has several limitations. First, the modest sample size from a single center may limit the generalizability of the findings. Second, the lack of detailed clinicopathological data, such as tumor stage, histological subtype, and smoking history, prevents subgroup analyses that could reveal important associations between biomarkers and specific disease characteristics. Third, the case-control design establishes association but not causation; longitudinal studies are needed to determine if these biomarker changes precede disease progression or are a consequence of it. Future research should address these gaps by prospectively enrolling a larger, well-characterized cohort.

## Conclusions

Lung cancer patients exhibited elevated oxidative stress and inflammation compared with healthy individuals, as evidenced by significantly increased CRP and NO levels and reduced SOD, GR, and GPx enzyme activities. These biomarkers may serve as supportive indicators for disease evaluation and therapeutic monitoring. Further research should explore their prognostic and predictive value in clinical settings.
